# Ruptured Pyogenic Liver Abscess as an Uncommon Cause of Pneumoperitoneum

**DOI:** 10.5334/jbsr.2784

**Published:** 2022-04-05

**Authors:** Olaia Chalh, Khaoula Sibbou, Jamal El fenni

**Affiliations:** 1 Military Hospital of Instruction Mohammed V, MA

**Keywords:** pneumoperitoneum, rupture, liver abscess, computed tomography

## Abstract

**Teaching Point:** Spontaneous ruptured gas-forming pyogenic liver abscess (GFPLA) is a life-threatening infection that mimics perforation of hollow viscous and need to be accurately diagnosed by computed tomography, which in turn helps to decrease the operative time and improve patient’s prognosis.

## Case History

**Figure 1:**
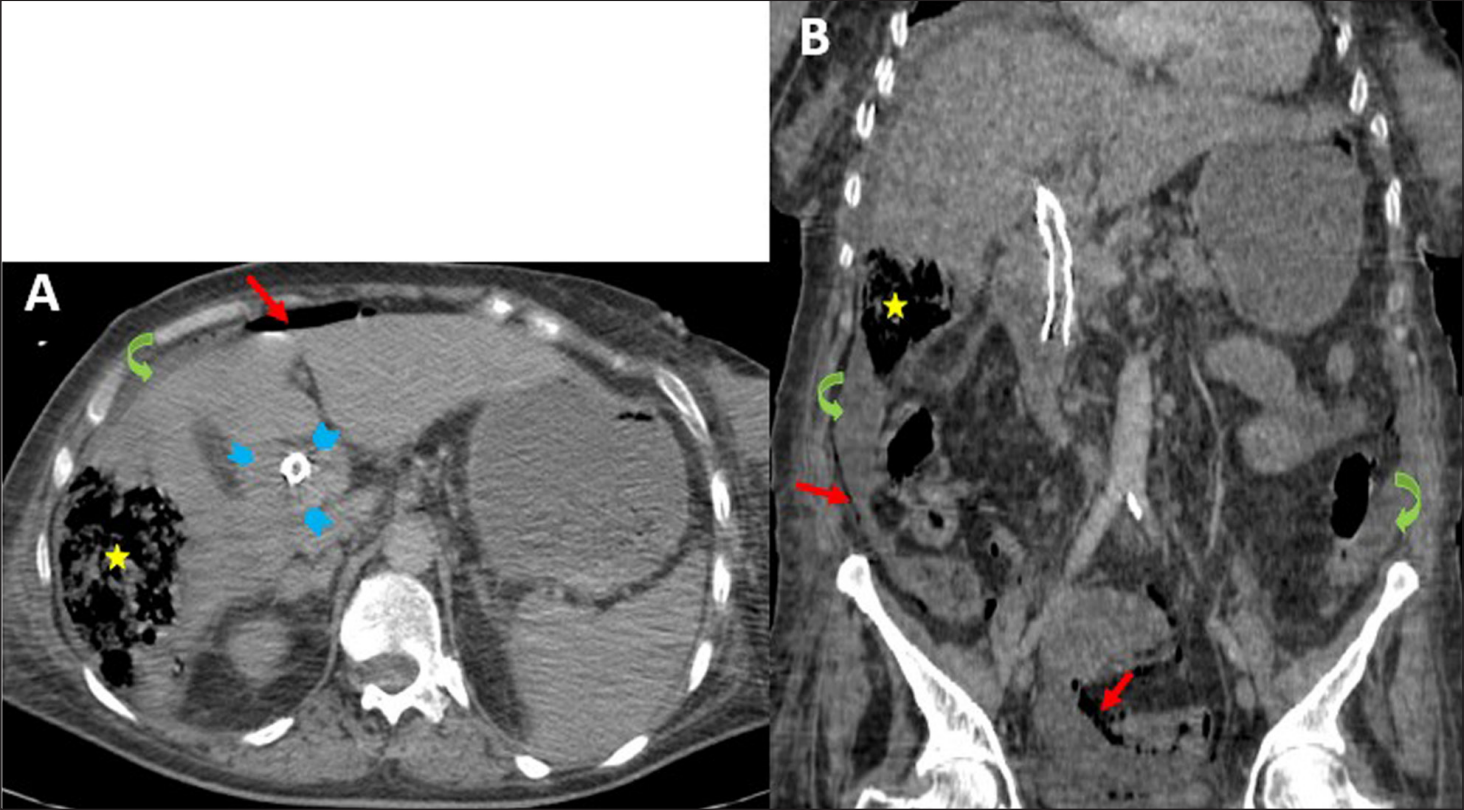


A 65-years-old woman with uncontrolled diabetes mellitus and history of endoscopic biliary stenting for choledocholithiasis three years ago was urgently admitted with complaints of severe abdominal pain and fever. Physical examination revealed high temperature of 39.5°C and generalized rigidity of the abdominal wall, suggesting peritonitis. Her heart rate was 120 beats/min and her blood pressure were 120/80 mmHg. She had a pallor and jaundice. Blood examination showed high levels of white blood cells, C-reactive protein, biomarkers of cholestasis, and serum glucose. Contrast-enhanced computed tomography (CT) of the abdomen was performed (***Figure 1A, B***). Axial and coronal images showed a gas-forming liver abscess (10 × 9 × 5 cm) at the segment VI adjacent to the capsule (asterisks), which was ruptured within the peritoneal cavity causing diffuse pneumoperitoneum (red arrows) with fat stranding and peritoneal effusion (curved arrows). Thickening and enhancement of common biliary duct wall, especially around biliary stent, suggestive of cholangitis was also noted (arrowheads). The patient passed away a few minutes after her admission into the surgery room.

## Commentary

Gas-forming pyogenic liver abscess (GFPLA) is a severe form of liver infection with a high rate of morbidity and poor prognosis especially in geriatric population. It occurs most commonly in patients with uncontrolled diabetes mellitus. *Klebsiella pneumoniae* is the most incriminate pathogen in the production of gas within the liver abscess [1].

Spontaneous rupture of GFPLA within peritoneal cavity causing pneumoperitoneum is a life-threatening complication. It occurs in almost 5.4% of all liver abscesses [1].

Clinically, it is often manifested as septic shock or sometimes asymptomatic in diabetic patients due to blunted immunological response [1].

Emergency imaging modalities play an optimum role in correct diagnosis and may contribute to treatment planning. Chest and abdominal radiography may reveal pneumoperitoneum with gas pockets on the right-upper abdomen. Ultrasound may show high-attenuating hyperechogenic foci into the liver. However, it is often surpassed by computed tomography (CT), which remains the key to an early and accurate diagnosis. As pneumoperitoneum is often due to perforation of hollow viscous, CT may rule out differential diagnosis and confirm the presence of gas within the liver abscess with signs of peritonitis [1].

In the absence of diffuse peritonitis, percutaneous or laparoscopic drainage of localized GFPLA can be performed successfully. However, when features of peritonitis exist, laparotomy with debridement and drainage of the abscess cavity or partial hepatectomy remains an indispensable options of surgical treatment [1].
